# A health system framework for perinatal care in South African district hospitals: a Delphi technique

**DOI:** 10.1186/s12913-019-4200-4

**Published:** 2019-06-20

**Authors:** Ntombifikile Maureen Nkwanyana, Anna Silvia Voce, Sthandwa Octavia Mnqayi, Benn Sartorius, Helen Schneider

**Affiliations:** 1 Discipline of Public Health Medicine, College of Health Sciences, University of KwaZulu-Natal, Durban, South Africa; 2 King Cetshwayo Health District Office, KwaZulu-Natal Department of Health, Empangeni, South Africa; 3 Faculty of Infectious and Tropical Diseases, London School of Hygiene and Tropical Medicine, London, UK; 4 School of Public Health and SAMRC Health Services to Systems Unit, University of the Western Cape, Cape Town, South Africa

**Keywords:** Health system framework, Health system strengthening, Perinatal care, Intrapartum care, Programme-specific interventions, South Africa, District hospitals

## Abstract

**Background:**

The majority of perinatal deaths occurring in low- and middle- income countries are preventable. South Africa is a middle-income country with consistently high perinatal mortality rates and most factors contributing to preventable deaths are linked to the functioning of the health system. Particularly of concern in South Africa is the high perinatal mortality in district hospitals, where most births occur and where intrapartum care is provided to women of low and intermediate risk. Therefore, it is crucial to strengthen the health system for perinatal care in district hospitals. There is currently no consolidated documented framework outlining contextual health system domains and indicators that are key to providing effective perinatal care in district hospitals. The purpose of this study was to derive key health system domains and indicators necessary to measure the performance of the health system for perinatal care in South African district hospitals.

**Methods:**

The Delphi technique was used in collecting data from a panel with experts drawn from disciplines connected with the functioning of the health system for perinatal care in South Africa. The study enrolled thirteen experts from whom data on key health system domains and indicators for perinatal care were derived. The project reference group gave guidance to the development of the framework and ascertained its relevance to the South African setting.

**Results:**

The Facility Based Health System Framework for Perinatal Care comprising domains and indicators necessary to measure health system performance in South African district hospitals was derived from data. The broad structure of the proposed framework aligns with the WHO Health Systems Framework. Each critical building block has detailed domains and indicators that illuminate essential facility-level and programme-specific elements that require attention for strengthening the health system for perinatal care.

**Conclusion:**

The proposed framework can enable district hospital management teams to identify gaps in the health system for perinatal care, which need to be strengthened in order to alleviate the burden of perinatal deaths in district hospitals.

## Background

High perinatal mortality remains a global public health concern. The majority of perinatal deaths occurring in low- and middle-income countries (LMIC) are preventable and most are intrapartum-related [1–4]. Although patient factors do contribute to intrapartum-related deaths, evidence shows that most factors contributing to preventable mortality are directly linked to the functioning of health systems [1, 3]. Specifically, in low resource settings, 66.7, 11.9 and 7.1% of preventable intrapartum-related deaths have factors relating to substandard care by a health worker, administrative factors, and referral factors respectively [1]. Consequently, there is a great need to strengthen the health system for perinatal care in LMIC in order to save lives [5].

South Africa is a middle-income country with consistently high perinatal mortality rates even among babies who are expected to have a good chance at survival [4]. The high prevalence of intrapartum-related deaths for babies who weigh at least 2500 g at birth clearly shows weaknesses in perinatal health care in South Africa [4, 6]. Essentially, in 2008 and 2009, approximately 35% of fresh stillbirths in South Africa weighed at least 2500 g, and in the 2010/2011 period, the reported intrapartum-related mortality for babies in this weight category was 6.37 per 1000 births [7, 8]. In one province in South Africa, the analysis of preventable perinatal deaths following an intrapartum event indicated that early neonatal deaths were more likely to have a healthy mother [9]. Such deaths, which cannot necessarily be predicted during antenatal care, and are not prevented during intrapartum care, reflect a need to focus on potential underlying causes; and these can be better understood by exploring the context in which these deaths occur.

South Africa’s public healthcare delivery system is organized into levels of care, providing perinatal care health services for low risk to high risk pregnancies and births [10, 11]. Low risk births are designated to be midwife-led, in clinics and community health centres (CHCs), which provide basic emergency obstetric care. Clinics and CHCs refer intermediate risk births to district hospitals which provide comprehensive emergency obstetric care [11]. High risk births are designated to be doctor-led in regional, tertiary and national central hospitals [11]. Overall, the healthcare delivery system has been designed to meet the healthcare needs of women during pregnancy, around the time of birth and after birth.

Perinatal mortality rates in South African district, regional, tertiary and national central hospitals were 30.5, 42.7, 55.3 and 64.3 per 1000 total births respectively in the 2012/2013 period [6]. The perinatal mortality in regional, tertiary and national central hospitals is presumably from high risk pregnancies and births with complications [11]. Although district hospitals have a lower perinatal mortality rate, the absolute number of perinatal deaths occurring in district hospitals is higher compared to other levels of care [6]. The high perinatal mortality in district hospitals is of concern as deaths are occurring in intermediate risk pregnancies [11]. The high number of deaths in district hospitals could be partially attributed to the quality of care, which as measured by the perinatal care index, is poorer [8, 12]. A survey conducted in 2012 indicated that the ability of district hospitals to provide lifesaving services is poor, implying that safe intrapartum care is not consistently available in district hospitals [13]. The inefficiencies in district hospitals could be resulting from a lack of resources and competencies to deliver comprehensive intrapartum care services [10, 13]. District hospitals account for approximately 41% of births in the public health sector [8]. Therefore, there is a great need to focus health system strengthening strategies on district hospitals in order to reduce perinatal mortality in babies born to intermediate risk mothers, toward improving perinatal outcomes.

The environment in which perinatal care is provided must be better understood before implementing health system strengthening strategies [14]. There is currently no consolidated documented framework outlining contextual health system domains and indicators that are key in providing effective perinatal care in South African district hospitals. Consequently, district hospital management teams are constrained in making evidence-based decisions in prioritizing areas of intervention for strengthening the health system for perinatal care. Hence, this study focused on deriving key health system domains and indicators, necessary to measure the performance of the health system for perinatal care in South African district hospitals. Understanding key contextual health system domains and indicators will in turn enable district hospital management teams to identify gaps in the health system for perinatal care, which need to be strengthened in order to alleviate the burden of perinatal deaths in district hospitals.

## Methods

### Aim of the study

The aim of the study was to develop a health system framework for perinatal care in South African district hospitals.

### Study design

The Delphi technique was used in collecting data from a panel of experts, comprising disciplines connected with the functioning of the health system for perinatal care in South Africa. The Delphi technique was implemented as it allowed an iterative process of data collection and analysis until consensus was reached on key health system domains and indicators for perinatal care [15].

### Study setting

Experts were based in two South African provinces, namely KwaZulu-Natal and Gauteng, but had a national perspective. The project reference group were based in two provinces in South Africa, namely KwaZulu-Natal and Western Cape, and had a national perspective. In-depth interviews were conducted in the experts’ workplaces, and consensus was reached through electronic communication.

### Sampling strategy and sample size

There were two groups of study participants: a group of experts and a project reference group.

#### Selection of experts

Thirteen experts, from whom data on key domains and indicators of a health system for perinatal care were derived, were purposively sampled to participate in the study. Experts were selected based on their expertise in perinatal care and/or on sound knowledge of the functioning of the health system for perinatal care in South Africa. A sample size of 10 is the recommended minimum for the Delphi technique [16]. The sample size of 13 was implemented to ensure maximum variation [17]. Table 1 presents background information of experts as well as the criteria that influenced their purposive selection.

**Table 1:** Background information of experts

Expert	Profession	Clinical experience in intrapartum and early neonatal care	Experience in providing outreach services to district hospital labour wards	Experience in managing health services for maternal and perinatal care at a district level	Involvement in research on South African maternal or perinatal care issues	Sound knowledge of the South African health system for perinatal care	Experience in training postgraduate students in obstetrics or neonatal care	Experience in district hospital management
1	Paediatric nurse	✓	✓	✓		✓		
2	Advanced Midwife	✓	✓	✓		✓		✓
3	Paediatric nurse	✓	✓			✓	✓	
4	Obstetrician	✓	✓	✓	✓	✓	✓	✓
5	District hospital clinical manager	✓				✓		✓
6	Obstetrician	✓	✓		✓	✓	✓	
7	District hospital nursing manager	✓			✓		✓	
8	District Hospital CEO	✓			✓	✓		✓
9	District Hospital Medical Manager	✓				✓		✓
10	Obstetrician	✓	✓	✓	✓	✓	✓	
11	Advanced Midwife	✓	✓	✓	✓	✓		
12	Neonatologist	✓		✓	✓	✓	✓	
13	Public Health Specialist	✓	✓			✓		

#### Selection of the project reference group

Four experts in disciplines related to the functioning of health system for perinatal care were requested to be part of the project reference group, which provided guidance to the development of the framework and ascertained its relevance to the South African setting. The group comprised one obstetrician and one paediatrician, who were respectively responsible for governance of the obstetric and paediatric services in one South African province. The third member was an advanced midwife with postgraduate training in public health and with previous experience in governing perinatal care issues at provincial level. The three members of the project reference group who had experience of governing perinatal care issues at provincial level had a nuanced understanding of health systems performance for optimal perinatal care, as policies for implementation are governed at provincial level in South Africa. The fourth member was a public health specialist who had a strong publication record in health systems research and was recruited to particularly contribute to the components and domains of the health system framework.

### Data collection and analysis

Data were collected and analysed in three rounds as described below.

#### Data collection and analysis – first round

In the first round of data collection, the principal investigator conducted face-to-face in-depth interviews with experts. A data collection tool, comprising open-ended questions, was developed by the principal investigator and a co-investigator who had solid experience in qualitative research and health systems research. Questions were formulated to elicit health system factors with either positive or negative influence on perinatal outcomes. The developed data collection tool was used to interview first two experts. Data obtained were transcribed and analysed by both the principal investigator and the same co-investigator. The data collection tool was modified based on the nature, quality and depth of data obtained in the preliminary interviews. Specifically, the enhanced data collection tool covered questions specific to each health system building block. An experienced transcriber was hired to transcribe recordings of the subsequent 11 expert interviews, and the analysis was done by the principal investigator and the same co-investigator.

Interview data was analysed using the directed content analysis approach, to identify codes and themes [18]. To ensure simplicity and comprehensiveness, the World Health Organisation (WHO) Health System Framework served as an organising framework for sorting codes and identifying major themes [19]. One member of the project reference group advised on the most correct formulation of domains and indicators based on South African guidelines for maternity care, and on the alignment between domains and building blocks [11, 19].

#### Data collection and analysis – second round

A list of all extracted domains and indicators, categorized by building blocks, was sent to twelve experts, as the thirteenth expert was lost to follow-up. Experts were requested to rate the impact of each indicator on perinatal care and perinatal outcomes. Out of twelve experts who were requested to provide ratings, one expert declined further participation in the study. Therefore, ratings were received from eleven experts. The rating scale ranged from zero to five with the connotation presented in Table 2.

**Table 2:** Rating Scale specifying perceived impact of health system indicators on perinatal care in district hospitals in South Africa

0- no impact	
1- very small or negligible impact	
2- small impact	
3- moderate impact	
4- large impact	
5- very high or profound impact	

Inclusion of domains and indicators in the health system framework for perinatal care was determined by assessing the percentage of experts who gave a rating of four or five, stipulating that an indicator had either large or profound impact on perinatal care and perinatal outcomes. Frequencies and percentages of the ratings were calculated by the principal investigator using Stata version 13. The guidelines informing inclusion of indicators are presented in Table 3.

**Table 3:** Guidelines informing inclusion of indicators in the health system framework for perinatal care in South Africa

Percentage of experts indicating large or profound impact	Decision
70% and above	Include
40 to 69%	Indeterminate
39% and below	Exclude

The results were discussed in a face-to-face meeting with all members of the project reference group who advised on relevance of the domains and indicators. In addition, the project reference group were provided with an opportunity to suggest additional domains and indicators, which they felt had been omitted through the previous round of data collection.

#### Data collection and analysis – third round

All indicators that were considered indeterminate in the second round, as well as additional indicators suggested by the project reference group were sent to eleven experts in the third round. At this stage, experts were requested to make a binary choice, and indicate whether they agree to include each indicator in the framework or not. All indicators that were deemed necessary to be included by at least 70% of experts were incorporated in the framework. Subsequently, all domains and indicators on which there was consensus were included in the framework. Table 4 presents the number of domains and indicators that were included in the framework in both the second and third rounds of data collection.

**Table 4:** Number of domains and indicators rated in the second and third rounds of data collection

	Second Round					Third Round*				Final	
Building Block	Domains sent for rating	Indicators sent for rating	Indicators			Indicators				Domains in the framework	Indicators in the framework
			Included	Excluded	Indeterminate	Suggested by project reference group	Total	Included	Excluded		
Health Information System	2	8	6	1	1	2	3	3	0	2	9
Health Financing	2	2	2	0	0	0	0	0	0	2	2
Health Workforce	9	29	24	3	2	2	4	0	4	9	24
Administrative factors	6	29	27	1	1	0	1	1	0	6	28
Leadership	8	29	25	2	2	2	4	3	1	8	28
Service Delivery	9	52	46	6	0	0	0	0	0	9	46
Total	36	149	130	13	6	6	12	7	5	36	137

*One domain with one indicator which was suggested by a member of the project reference group was deemed not necessary for inclusion in the framework

## Results

The domains and indicators for health system performance for perinatal care derived from the three rounds of the Delphi technique are presented in Tables 5, 6, 7, 8, 9 and 10 below, aligning to the building blocks contained within the WHO Health System Framework. Specifically, the six building blocks are labelled as Leadership, Health Information System, Health Workforce, Medical Resources and Infrastructure, Health Financing and Service Delivery.

**Table 5:** Leadership for perinatal care: domains and indicators

Domains	Indicators
Accountability	1. Operational managers have documented disciplinary procedures for control and prevention of the following: a) Late coming b) Absenteeism c) Non-adherence to clinical guidelines d) Lack of respect or abusive behavior by health care professionals toward mothers
	2. Operational managers meet, within 24 h, with the team of health care professionals who were on duty during an adverse perinatal event (death or morbidity) resulting from intrapartum care
	3. Operational managers have clear standard operating procedures for implementing a perinatal audit process (i.e. 24-h meeting post perinatal death or morbidity; preparatory meeting for monthly review meeting; and monthly perinatal review meeting)
Protocols of management	Operational managers have documented protocols with regards to the following: 1. Clear referral protocol from district hospital to regional hospital
	2. Clear referral protocol from clinic or Community Health Center (CHC) to district hospital
	3. Clear protocol with admission and discharge criteria for mothers
	4. Clear protocol with admission and discharge criteria for neonates
	5. Clear protocol, guided by staff norms, on staff allocation per shift, for latent phase and labour wards, with respect to number of staff
	6. Clear protocol, guided by staff norms, on staff allocation per shift, for latent phase and labour wards, with respect to health care professional skill mix
	7. Clear protocol on maintenance of medical equipment
	8. Clear protocol on procurement of medical equipment
	9. Clear protocol on monitoring of medicines stock
	10. Clear protocol on infection prevention and control
	11. Availability of protocols for intrapartum care (as per Maternity Care Guidelines)
	12. Availability of protocols for neonatal care (as per Initiative for Newborn Care (INC) guidelines)
Supervision	1. Operational managers ensure ongoing supervision for all staff in latent phase and labour wards
	2. Operational managers ensure clinical mentorship is provided to all health care professionals in latent phase and labour wards whose perinatal care skills need improvement
Induction Program	1. Operational managers allocate an advanced or experienced midwife to work alongside a health care professional who has just completed nursing training and has never worked in latent and labour wards
	2. Operational managers ensure that experienced health care professional who have not been working in latent and labour wards go through an induction program to review and assess all important processes and skills for perinatal care
	3. Operational managers ensure that skills of new staff (newly qualified and those who have been working in other hospital units) in latent and labour wards are assessed before and after the induction program
Teamwork	Hospital clinical manager ensures that doctors and nurses who work in latent phase and labour wards, work in harmony
Interest in perinatal outcomes	Senior hospital managers consistently participate in monthly perinatal review meetings that are held in their district hospitals
Stability	1. District hospital has a formally appointed hospital CEO (not an Acting CEO)
	2. Minimal management turnover (i.e senior hospital managers remain in leadership posts for at least 3 years)
Community Engagement	Senior hospital managers organize campaigns to engage with community to raise awareness on risk factors for perinatal deaths that are attributable to patient factors

**Table 6:** Health Information System for perinatal care: domains and indicators

Domains	Indicators
Processing of data	1. Use of admissions and discharge register in compiling midnight statistics
	2. Use of birth register in compiling midnight statistics
	3. Operational manager and health care professionals in labour wards interrogate (check accuracy, analyze and interpret) perinatal data before it is sent to the Facility Information Officer (FIO) for capturing
	4. Midnight statistics sent to the FIO for capturing daily
	5. Monitoring and evaluation manager interrogates (checks accuracy, analyses and interprets) perinatal data before it is captured in the District Health Information System (DHIS)
	6. Operational manager of labour ward participates in monthly district hospital information meeting
	7. Operational manager of labour ward adheres to data submission timelines
Use of data	1. Health care professionals and operational managers in labour wards analyse data specifically for hospital use, not only for reporting
	2. Operational manager in labour ward uses data to make decisions regarding routine operation of wards

**Table 7:** Health Workforce for perinatal care: domains and indicators

Domains	Indicators
Skill mix ratio	1. Availability of the following staff in labour wards guided by staff norms, per shift. a. Advanced midwives b. Midwives c. Enrolled nurses d. Doctors
	2. Availability of an advanced midwife in every shift
Number of health care professionals	1. Availability of the following staff in labour wards, with respect to staff numbers guided by staff norms, per shift. a. Advanced midwives b. Midwives c. Enrolled nurses d. Doctors
	2. Minimal staff turnover for advanced midwives, midwives, doctors and enrolled nurses per year
Clinical mentorship	All new staff (i.e. newly qualified and those who have been working in other hospital units) in latent phase and labour wards are allocated a clinical mentor until they are competent to provide perinatal care
Training	1. Per shift in latent phase and labour wards, there is at least one midwife or advanced midwife who has participated in all Essential Steps in the Management of Obstetric Emergencies (ESMOE) drills
	2. Per shift in latent phase and labour wards, there is at least one midwife or advanced midwife who has attended the Helping Babies Breathe (HBB) drill
	3. Per shift in neonate wards there is at least one midwife, advanced midwife or enrolled nurse who has attended the Initiative for Newborn Care(INC) training
	4. All staff allocated per shift have attended training on the proper use of medical equipment
Drills	1. All ESMOE modules are covered every month in labour ward drills 2. HBB drills occur monthly in labour ward
Staff rotation	At least 50 % of staff in labour and neonate units are non-rotating over one year
Teamwork	1. Doctors and nurses conduct ESMOE drills together
	2. Doctors and nurses conduct HBB drills together
	3. Doctors and nurses doing hospital rounds together
	4. Advanced / experienced midwives work co-operatively with doctors in clinical decision making for perinatal care
Anchor doctor	In hospitals that have less than 300 births per month, the availability of a doctor who is primarily responsible for the maternity unit, but also services other hospital units in the hospital
Dedicated doctor	In hospitals that have at least 300 births per month, availability of a doctor who is solely responsible for the maternity unit on a full-time basis

**Table 8:** Medical Resources and Infrastructure for perinatal care: domains and indicators

Domains	Indicators
Medicines	1. Availability of signal drugs (i.e. parenteral antibiotics, parenteral uterotonic drugs and parenteral anticonvulsants for pre-eclampsia and eclampsia)
	2. Availability of essential medicines for perinatal care in wards (as per essential drug list)
	3. Proper storage of medicines, (i.e. in a cool environment)
	4. Monthly stock taking of general medicines
	5. Weekly stock taking of dependency producing drugs (schedule 5 and above)
Medical Equipment	1. Availability of adequate numbers and functional medical equipment
	2. Availability of back up medical equipment for use while some stock has gone for repairs or servicing
	3. Availability of emergency boxes for emergency intrapartum care
	4. Availability of resuscitation trollies for emergency neonate care
Functional Theatre	1. Functional theatre 24 h, 7 days a week
	2. Availability of essential theatre drugs (as per essential drug list)
	3. Availability of functional essential theatre equipment
	4. Availability of backup theatre equipment for use while some stock has gone for repairs or servicing
	5. Availability of functional air-conditioning
	6. Availability of back-up generator
	7. Availability of the following theatre staff with respect to staff norms per shift a. Operating theatre nurse b. Enrolled nurse
Obstetric Ambulances	1. Access to obstetric ambulance on site
	2. Waiting time for obstetric ambulance to transfer patient to regional hospital less than one hour
	3. Availability of functional obstetric ambulance equipment
	4. Availability of skilled birth attendant during transit
	5. Availability of essential medicines for intrapartum care during transit
Mothers’ waiting homes (for rural hospitals)	1. Availability of mothers waiting homes on site
	2. Guidelines for admission to waiting homes followed
	3. Adequate utilization of mothers’ waiting homes
Infrastructure	1. Location of maternity ward and neonate high care unit in close proximity to each other
	2. Availability of necessary power points to connect equipment for perinatal care (i.e. suction points and oxygen points.)
	3. Availability of Kangaroo Mother Care ward

**Table 9:** Health Financing for perinatal care: domains and indicators

Domains	Indicators
Procurement Plans	Operational manager in labour ward submits procurement plans annually
Adequacy of funds	Maternity unit has adequate funds to purchase medicines, medical equipment and hire adequate and diverse health care professionals

**Table 10:** Service Delivery for perinatal care: Domains and indicators

Domains	Indicators
Packages of Care	Implementation of the following packages of care: 1. Basic Antenatal Care
	2. Basic intrapartum care
	3. Comprehensive Emergency Obstetric Care
	4. Initiative for Newborn Care
	5. Postnatal care
	6. Kangaroo Mother Care
Referral System	1. Use of Situation Background Assessment Recommendation (SBAR) in referring patients
	2. Effective communication between labour ward and referring clinic or CHC at both management and health care professional level
	3. Effective communication between district hospital labour ward and regional hospital labour and neonate wards at both management and health care professional level
	4. Providing feedback on patient progress to referring clinic or CHC
	5. Receiving feedback on patient progress from regional hospital labour and neonate wards
Continuity of care	1. Handover between shifts consistently done per patient
	2. Standardized handover between EMRS (obstetric ambulance) and labour ward
	3. Standardized handover between EMRS (obstetric ambulance) and neonatal ward
Clinical guidelines	1. Accessibility of clinical guidelines for intrapartum care
	2. Accessibility of clinical guidelines for neonatal care
	3. Display of guidelines for emergency intrapartum care
	4. Display of guidelines for emergency neonatal care
	5. Adherence to intrapartum and neonatal care clinical guidelines
Responsiveness to patient needs	1. Cleanliness of maternity and neonate wards
	2. Cleanliness of medical equipment for perinatal care
	3. Patient satisfaction with regards to care received from health care professionals
Outreach Program	1. Obstetrician from regional hospital visit labour ward monthly to provide outreach services (i.e. auditing of quality of care, auditing of files, on-site training, ward rounds, emergency drills, etc.)
	2. Pediatrician from regional hospital visit labour ward monthly to provide outreach services (i.e. auditing of quality of care, auditing of files, on-site training, ward rounds, emergency drills, clinical care, etc.)
	3. Members of District Clinical Specialist Team (DCST) team visit labour
	4. ward at least once a month to provide outreach services (i.e. auditing of quality of care, auditing of files, on-site training, ward rounds, emergency drills, clinical care, etc.)
	5. Telephonic clinical support from specialist in regional hospitals
Perinatal Audit Process	1. Preparatory meeting for perinatal mortality and morbidity review meeting occur monthly
	2. Perinatal review meetings occur monthly
	3. Perinatal review meetings result in clear action plans
	4. Action plans are followed up in the subsequent meetings
	5. Impact of decisions taken are evaluated and discussed in perinatal review meetings
	6. The following stakeholders attend perinatal review meetings a. Hospital manager b. Medical OR Clinical Manager c. Nursing manager d. Assistant nursing manager (responsible for maternity and paediatric units) e. Labour ward operational manager f. Primary Health Care (PHC) Manager based in district hospital g. CHC or Clinic operational managers h. Doctors working in labour unit i. Advanced midwives j. Midwives k. Community care givers (CCG) facilitator
Audits	1. Health care professionals and operational manager audit adherence to guidelines for intrapartum and neonate care
	2. Perinatal mortality and morbidity are audited by health care professionals and operational managers in hospital together with DCSTs at least four times a year
Staff attitude	1. Health care professionals treat patients with respect
	2. Health care professionals having respect towards their fellow colleagues irrespective of professional rank or discipline

### Leadership

Leadership is defined as the role of both senior hospital managers as well as labour and neonatal ward managers in ensuring that the health system for perinatal care functions optimally. Leadership domains and indicators of health system performance, which by consensus were considered to have a major impact on perinatal mortality, are presented in Table 5.

Most indicators in the leadership building block are responsibilities of operational managers at ward level. However, the impact of senior hospital managers on perinatal outcomes was emphasised. Apart from ensuring availability and implementation of policies, both hospital and ward level operational managers are responsible to oversee overall functioning of the health system for optimal perinatal care. In recognition of the role of senior hospital managers in perinatal care, the Department of Health authorities have included ‘ensuring improved perinatal outcomes’ in the hospital managers’ key results areas (KRA).

*“It* [improved perinatal outcomes as part of hospital manager’s key results area] *has now been instituted by Minister of Health. They* [the CEOs] *had orientation regarding maternal and perinatal outcomes*. *… Based on the recommendations*, *they* [improved perinatal outcomes] *have been put into the KRAs* [Key Result Areas] *for the CEOs. So they* [the CEOs] *are directly responsible.”*[KI-01].

In addition to ensuring optimal functioning of the health system for perinatal care, hospital managers should engage with communities concerning potential risk factors for perinatal deaths.

*“….and if they* [the CEOs] *have picked up problems, they must communicate with the community; and say ‘Guys, you come in late, that’s why we’ve got so many perinatal deaths’. So that is the responsibility of the manager*.” [KI-12].

*“If you ignore the community, then maybe you must start forgetting about the whole thing* [improved perinatal outcomes]. *Honestly.”* [KI-03].

### Health information system

The Health Information System building block focuses on routine data management processes as well as the use of data for decision-making. The processes include capturing, sorting, analysing and sharing knowledge amongst stakeholders. Domains and indicators of health information system at ward level and at district hospital level are presented in Table 6.

Experts highlighted that the value health care professionals attach to data has great influence on the quality of the data entered in source documents. Health care professionals are primarily trained to provide clinical care. As a result, the importance of health systems related data might not be apparent, more especially to newly qualified professionals. Ensuring that all health care professionals appreciate the importance of health systems related data, and its impact in improving quality of care and facilitating proper decision-making, was emphasised.

*“If you are talking about a new nurse, a new midwife, data is the last thing on her mind…. Even myself as a new nurse back then, I didn’t appreciate data.”* [KI-01].

“*And the nurse has to know how important the data are, because without data we don’t know where we’re going*.” [KI-04].

### Health workforce

In this study, health workforce refers to health care professionals who provide perinatal care. Several domains and indicators were identified, relating to the availability and competence of the health workforce, and are presented in Table 7.

The skill mix ratio, the number of health care professionals, and the availability of an anchor or a dedicated doctor are the domains identified to ensure the availability of an adequate and diverse health workforce to provide perinatal care. Clinical mentorship, training, drills and staff rotation are domains identified that reflect competence of the health workforce. A few critical challenges emanated from respondents, which were described as constraining the availability and competence of the health workforce in district hospitals. For instance, some rural district hospitals have a challenge of retaining skilled doctors and midwives who migrate to urban settings for various reasons, including uncomfortable accommodation. Some doctors migrate to regional hospitals as they feel they get limited exposure with respect to the scope of clinical services they offer in district hospitals. The challenge of high staff turnover, more especially in rural district hospitals, has a potential to leave district hospitals with less competent staff. In addition, it may affect the quality of service delivery, as health care professionals become overwhelmed by high workloads.

*“On a whole, yes they are competent, but that competent staff member, as I am speaking, she is handing in her resignation letter… The hospitals send them* [clinical staff] *for training, but they are only committed to stay for that certain period that they have signed in the contract. After that, they go. It’s now become a culture, which, if one advanced midwife comes back* [from training]*, the other one leaves.”* [KI-01].

“*I spent some time at a nurses’ workshop a month ago… The big issue was burnout. Nurses are feeling burned-out and not supported. She is frustrated and burned-out, and one of the things when you are burned-out, and I have experienced it myself, you stop caring.”* [KI-04].

In addition, teamwork amongst health care professionals was specified as an important domain for the performance of the health workforce. The main challenge mentioned was the lack of collaboration between doctors and nurses, particularly in conducting joint ESMOE and HBB drills, as well as in joint clinical decision-making.

*“And the doctors sometimes don’t listen to the nurses because they say, ‘No, you’re a nurse, you are supposed to listen to me. It’s not* [about] *me listening to you.’ I think that is wrong. So part of teamwork is that this patient that you’re looking after, both of you are equally responsible. So if I see something as a nurse, I should be able to say, ‘Doctor, this patient has got this, I think we should do this’. The doctor must not see that as a threat. And the same thing with a nurse, she should be able to listen to the doctor and respond appropriately. So what you see sometimes is that because the person is a doctor, he comes in as an intern, he’s just got one year out of school and a nurse has been there for thirty years, who’s got experience. All of a sudden, because he is a doctor, the name is ‘doctor’; she* [the nurse] *must listen to this guy with one-year experience. So to me something is very wrong.”* [KI 12].

### Medical resources and infrastructure

The medical resources and infrastructure building block focuses on the availability of essential medicines, medical equipment, transport services and infrastructure. Basically, it measures the “hardware” of the health system for perinatal care [20]. The identified domains and indicators that must be monitored to ensure optimal perinatal care are presented in Table 8 below.

Experts strongly emphasised the consistent adequate supply of medicines for perinatal care. Reportedly, district hospitals have shortages of functional medical equipment, exacerbated by a tiresome procurement and replacement process. However, one expert believed that intrapartum care medical equipment is mainly important for mothers who experience complications during labour. However, health care professionals tend to over-rely on medical equipment for low-risk births in district hospitals.

*“Again, going back to the fact that you can labour at home and deliver a very good nice bouncing baby. That’s how we were made. We were made to deliver properly. But* [when] *in labour, then it’s* [important] *to monitor* [the labour]. *That’s why we have tools to monitor the labour; the partogram was made to identify a woman who struggles to follow the normal route.”* [KI-06].

### Health financing

Health financing mainly refers to the role played by operational managers at ward level in ensuring that the wards that offer perinatal care have adequate resources. South African district hospitals offer free health services to mothers and neonates, and government financing is the only stream of funds for healthcare services [21]. Domains and indicators for health financing for perinatal care are presented in Table 9.

Apparently, hospital managers do not have full control on the total funds allocated to their hospitals. Funds are directly allocated to the entire hospital and the distribution of funds among the diverse services offered by the hospital is at the discretion of senior hospital managers.

*“For a district hospital, there are many pressures, you* [a hospital manager] *could have a pressure of diverting resources to where you* [a hospital manager] *have the biggest burden* [of mortality and morbidity]*… and adults always take priority* [of funds allocation]*.”* [KI-13].

*“District hospital managers contribute to what their budget allocation is, but they do not have final control on their allocation… They do not always get that which they ask for because everything is always limited to budget… That means they do not have final control on human resources as the money they get is used to pay for everything within their delegation.”* [KI-13].

### Service delivery

Service delivery in this study refers to a process through which health care professionals, health programmes and policies are coordinated and implemented, to provide optimal perinatal care with the aim to minimized perinatal deaths. Key domains and indicators of service delivery are presented in Table 10.

The variety of domains and indicators relating to service delivery show that it is not enough for the labour and neonatal wards to offer the essential packages of care, but the manner and process of providing care is important. Of note is the involvement of senior hospital managers in ensuring optimal delivery of perinatal care services. Senior hospital managers are expected to be involved in the perinatal audit process; this also reflects their invaluable influence on perinatal outcomes.

“*The position you are holding, the responsibility* [for accountability] *that is on you, makes people comply, without even saying anything, just by being there….. It gives* [credibility to] *the meeting. But the CEOs were not participating that much. Again, the decisions, it* [availability of the CEOs in perinatal review meetings] *also creates an opportunity for thorough decision-making. We will not need to have another forum meeting because the person who needs to make a decision is there. Actually, that person* [the CEO] *is the chairperson of the cash flow, if we need money to do something. Obviously, when we put a motivation, its going to be approved. You have already agreed with him in the meeting. It makes a lot of difference.”* [KI-02].

Essentially, the essence of strengthening health systems is to improve the quantity and quality of service delivery. Any weaknesses in the other building blocks of the health system eventually disadvantage service delivery. Thus, it is important that every actor in the health system play their role fully and continually, in order to alleviate the burden of perinatal deaths in South African district hospitals.

## Discussion

The key contribution of this study has been to provide the Facility Based Health System Framework for Perinatal Care (FBHSF-PC) comprising domains and indicators that are necessary to measure health system performance for perinatal care in South African district hospitals. Most health system frameworks that have been developed to date are applicable at national level, and have not been translated for implementation at facility level [19, 22–25]. However, managers at facility level have a strong influence on health system functioning and on how policies are implemented and hence experienced by health care professionals and the communities they serve [26]. Moreover, the majority of existing health system frameworks are not designed for programme-specific health system strengthening interventions [19, 22, 27]. Further, no consensus exists on what are programme-specific health system strengthening strategies to deliver optimum programme-specific interventions [23, 28]. Therefore, a framework to guide facility-level and programme-specific health system strengthening interventions is crucial.

Health facilities are at the frontline of service delivery, therefore their functionality has direct influence on health outcomes, which are the best measure of health system performance [29]. Since health facilities are the most peripheral organisational units within a decentralised health system, developments at facility level should accrue improvements at national level. Substantial progress has been noted globally in the delivery of health related interventions [19]. However, performance indicators aggregated at national level hide peripheral variation in performance, and therefore hide poor outcomes for the most vulnerable groups [19]. Focusing health system strengthening interventions at facility level will enable increased access to quality health services even for the most vulnerable groups in communities. Subsequently, the mandate of health systems to deliver efficient quality services to all people would be achieved [5, 19].

Programme-specific health system strengthening interventions have been shown capable to improve targeted health outcomes [23, 30]. For instance, application of health system strengthening interventions pertaining to maternal, neonatal and child health in several African countries had definite improvements on maternal and newborn health outcomes [23, 30]. Remarkably, programme-specific health system strengthening have a great potential to strengthen the overall health system as interventions are implemented within a broader health system [23, 31]. Since the functionality of a health system is evident in its capacity to deliver quality services for priority health issues, it is important to focus interventions to strengthening health systems for perinatal care, as maternal and newborn care are global health priority issues [5, 27, 32].

The broad structure of the FBHSF-PC aligns with the WHO Health Systems Framework which was used as an organising schema [19]. The alignment of FBHSF-PC to the WHO Health Systems Framework ensured that all critical health system building blocks previously identified as contributing to strengthening health systems are incorporated [19, 29]. The detailed domains and indicators within each critical building block of the FBHSF-PC illuminate essential facility level and programme-specific elements requiring attention for optimal perinatal care.

The FBHSF-PC has four additional components that are not featured in the WHO Health Systems Framework. These are: a) community engagement; b) teamwork amongst health care professionals; c) staff attitude; and d) the role of health facility managers in service delivery. Essentially, consistent engagement with communities with regard to health promotion and disease prevention is considered an effective and sustainable way to ensure improved health outcomes [22]. Hence, the Framework for Strengthening Health Systems to Improve Maternal, Neonatal and Child Health Outcomes and the Ouagadougou Declaration on Primary Health Care and Health Systems in Africa reflected community engagement as a major priority in the functioning of a health system [22, 23]. Effective teamwork amongst health care professionals is key for ensuring effective and safe patient care, and particularly for perinatal care, as cooperation from multiple disciplines is necessary to provide effective quality care [11, 33, 34]. Poor staff attitude is one of the barriers to health systems providing equitable and respectful health care, and is mainly experienced in public sector health facilities [30, 35, 36]. In South Africa, poor attitude of health care professionals towards mothers has been experienced routinely [10, 26, 37]. Hence it has been considered a key factor for maternal health in South Africa [25]. It is therefore necessary for managers at facility level to monitor staff attitude in order to ensure that mothers are treated with respect. The role of senior facility managers in the FBHSF-PC is not constrained to governance and supervision, as is the case with WHO Health Systems Framework and the World Bank Control Knobs Framework [19, 24]. It includes involvement in the perinatal audit process which has a direct impact on quality of services offered [38]. The four additional components included in the FBHSF-PC are crucial, as they strengthen the functioning of the health system at facility level.

The FBHSF-PC aligns well with the essential packages of care and the Guidelines for Maternity Care published in 2015 by the Ministry of Health in South Africa [11]. Therefore, application of the FBHSF-PC would be a first attempt to integrate health system strengthening with best practice clinical guidelines shown to be effective in the management of maternal and newborn health [39]. Therefore, the FBHSF-PC can become an effective and relevant mechanism to guide facility and unit managers in managing perinatal care in South African district hospitals.

The FBHSF-PC has been particularly designed to strengthen the health system for perinatal care for births occurring within a health facility. Thus, it does not address antenatal care challenges and patient related factors that are mainly associated with pre-admission perinatal deaths. Approximately 1.6% of women present with absent foetal heart on admission in South Africa [40]. Thus, implementation of the FBHSF-PC will ensure improvement of the performance of a health system in a majority of women who have live foetuses on admission.

The FBHSF-PC was validated during the course of its development. Specifically, face validity was assured through participation of the project reference group in ascertaining relevance of domains and indicators to the South African setting [41]. In addition, content validity was ensured by giving members of the project reference group an opportunity to suggest additional domains and indicators that were omitted by experts [41].

Through a Delphi technique, the FBHSF-PC identifies domains and key indicators to measure health system performance for perinatal care. The framework requires implementation for further refinement, which will involve an analysis of measurement challenges encountered. The FBHSF-PC does not measure the effect of critical building blocks and of overall health system performance on perinatal outcomes. Since the essence of strengthening health systems performance is to ensure improved health outcomes, there is a need to translate the FBHSF-PC to a model that will measure the impact of the identified building blocks on overall performance of health system, and in turn on perinatal outcomes [19, 29]. That is, criterion validity of the FBSHF-PC needs to be determined [41]. In addition, since a health system is a set of interconnected elements that function cooperatively, interactions between critical building blocks must be defined [29]. Thus, research on the application of the FBHSF-PC must be conducted to ascertain its utility and to develop a model that will assist managers to recognise areas of intervention in the health system that will yield greatest improvements in perinatal outcomes.

## Conclusions

Programme-specific health system strengthening interventions applied at facility level can yield optimum health outcomes. The proposed framework can empower district hospital management teams to identify gaps in the health system for perinatal care that need to be strengthened in order to alleviate the burden of perinatal deaths in district hospitals.

## Data Availability

The datasets analysed during the current study are available from the corresponding author on reasonable request and with permission of experts.

## Abbreviations

CEO: Chief Executive Officer
CHC: Community Health Centre
DCST: District Clinical Specialist Team
DHIS: District Health Information System
EMRS: Emergency Medical Rescue Services
ESMOE: Essential Steps in the Management of Obstetric Emergencies
FBHSF-PC: Facility Based Health System Framework for Perinatal Care.
FIO: Facility Information Officer
HBB: Helping Babies Breathe
INC: Initiative for Newborn Care
KRA: Key Result Areas
LMIC: Low- and middle-income countries
PHC: Primary Health Care
SBAR: Situation Background Assessment Recommendation
SDGs: Sustainable Developments Goals
WHO: World Health Organization
